# A Classification System to Detect Congestive Heart Failure Using Second-Order Difference Plot of RR Intervals

**DOI:** 10.4061/2009/807379

**Published:** 2010-03-21

**Authors:** R. A. Thuraisingham

**Affiliations:** 1A, Russell Street, Eastwood NSW 2122, Australia

## Abstract

A classification system to detect congestive heart failure (CHF) patients from normal (N) patients is described. The classification procedure uses the *k*-nearest neighbor algorithm and uses features from the second-order difference plot (SODP) obtained from Holter monitor cardiac RR intervals. The classification system which employs a statistical procedure to obtain the final result gave a success rate of 100% to distinguish CHF patients from normal patients. For this study the Holter monitor data of 36 normal and 36 CHF patients were used. The classification system using standard deviation of RR intervals also performed well, although it did not match the 100% success rate using the features from SODP. However, the success rate for classification using this procedure for SDRR was many fold higher compared to using a threshold. The classification system in this paper will be a valuable asset to the clinician, in the detection congestive heart failure.

## 1. Introduction

 The need to reach remote, underserved communities with life saving health care is an important area that warrants attention. Fast electronic communication and reliable automated classification systems will enhance this area of health care. In particular when cardiologists serving remote areas are few, reliable automated classification systems will not only free offsite cardiologists from routine visual analysis of electrocardiogram (ECG) data but also provide valuable specialized treatment to patients in remote areas from more experienced cardiologists elsewhere via electronic communication. The analysis and classification of large amount Holter monitor data is an aspect which is amenable to reliable automation. This paper concerns itself to develop such a system to distinguish congestive heart failure (CHF) subjects from normal (N) subjects. The patient measurements that are used for this automated analysis are the Holter monitor RR interval data. In the system that is being studied, these RR intervals are used in the construction of a second-order difference plot (SODP), whose features are then used as input in the classification algorithm. In addition the paper also looks at the results that are obtained using the standard deviation of RR intervals (SDRR) in the classification algorithm. 

Several attempts in this direction of automatic classification of CHF patients have been made with varying success. The use of power spectral densities of the RR intervals derived from autoregressive moving average (ARMA) with the artificial neural network (ANN) [1] is one of them. This gave a success rate of 83.3% in correctly classifying CHF patients. The sample set used here was limited, consisting of 12 N and 12 CHF subjects. In another approach detrended fluctuation technique along with unassisted K- means clustering analysis [2] was used. The success rate in the classification of CHF patients here was 86.7%. The most successful study involved the use of wavelet coefficients obtained from a discrete wavelet transform and multiclass support vector machines (SVM) with error correcting output codes (ECOC) [3]. The success rate here was 98.61%. One of the drawbacks of this procedure is the selection of a suitable kernel function which appears to be a trial-and-error process. One would not know the suitability of a kernel function and performance of the SVM until one has tried and tested with representative data. 

The method presented in this paper is an alternate procedure to the methods mentioned above. It uses time domain information and it is easy to implement compared to the above methods. A reliable automated system in the future can be a fusion of the both time domain and a successful frequency domain method. In this respect, the method presented here can not only be used to provide an independent classification tool, but also be used as a complementary method to verify the frequency domain results. 

In the Poincare plot, a technique taken from nonlinear dynamics, each sample value *x*(*n* + 1) is plotted against *x*(*n*) in a time series [4]. It displays the correlation between consecutive values in the time series. In the second-order difference plot (SODP) [5], (*x*(*n* + 2) − *x*(*n* + 1)) is plotted against (*x*(*n* + 1) − *x*(*n*)). It is a plot of successive rates against each other. The graph displays the correlation between consecutive rate values in a time series. In this paper the focus is on SODP. It is a simple analytical technique which could be a valuable additional tool in the analysis of heart rate variability (HRV). The objective of this paper is to examine the potential of this additional tool in the analysis and classification of cardiac RR interval data collected via Holter monitors, in particular to identify congestive heart failure patients (CHF) from normal (N) patients. 

Quantitative characterization of the Poincare plot to capture summary descriptors shows, that they are all related to linear aspects of the RR interval such as standard deviation of the RR intervals and the standard deviation of the successive differences [4]. There are no new HRV measures. On the other hand the second-order plot is characterized by a central tendency measure (CTM) [5] which is not related any linear HRV measure. It is likely to be measuring independent nonlinear information on the intervals [4]. However in this paper we do not limit the characterization of SODP to CTM only but include other features not discussed previously. These features are then used in a classification algorithm and statistically evaluated to determine whether the subject is N or has CHF.

Cardiac data used in this analysis comprises of RR intervals of N and CHF patients. Holter monitors often collect heart beat data over a 24-hour period. A fast and effective analysis of such data is valuable to the clinician. The second-order difference plot is amenable for such an analysis. In this paper features used to characterize these plots are studied, and its potential to separate healthy from diseased patients is illustrated. 

Heart rate variability analysis has shown much promise to predict heart disease. Patients with atrial fibrillation have RR intervals randomly distributed. Depressed heart rate variability is a predictor of patients with chronic and stable congestive heart failure [6, 7]. It has also shown to be a predictor of mortality in hospitalized patients with decompensated CHF [8]. Analysis of HRV involves evaluation of variables both in the time domain and in frequency domain. One of the common time domain variable computed is the standard deviation of RR intervals (SDRR). In this paper, the results using SODP are compared with SDRR using the same classification system. This is done for a data set that comprises RR intervals of 36 normal and 36 CHF patients.

## 2. Method and Materials

### 2.1. SODP of Cardiac RR Interval Data

**Figure 1:**
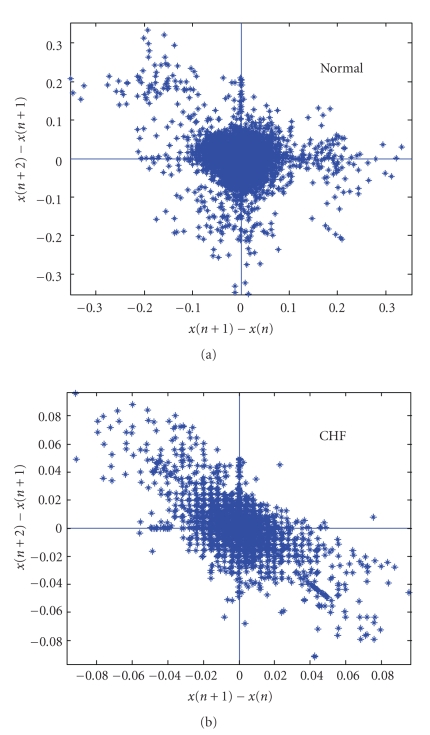
Second-order difference plots of a normal (a) and CHF (b) patient. Note the different scales in the two plots. Different scales were chosen to show clearly the structure in the two plots.

Figure 1 shows the SODP of cardiac RR data of a normal and a CHF subject. The data was obtained from MIT-BIH Normal Sinus Rhythm database and BIDMC Congestive Heart Failure database posted on Physionet [9]. The two RR interval time series used in this analysis was preprocessed [10] by the removal of trends and ectopic beats. Thirty thousand RR intervals were used for the normal and CHF subject. The plot clearly indicates differences between them, where, compared to a normal patient, the CHF patient has a lower variability. 

### 2.2. Measures to Quantify the Variability in SODP

In this section some measures to quantify variability in SODP are discussed. 

#### 2.2.1. Central Tendency Measure (*C* *T* *M*(*r*))

Central Tendency Measure (CTM(*r*)) [5] is a parameter that has been adopted to quantify the degree of variability in a second-order difference plot. The CTM is computed by selecting a circular region of radius *r*, around the origin, counting the number of points that fall within the radius, and dividing by the total number of points. Let *n *be the total number of points and *r *the radius of the central area. Then,

(1) $$\begin{array}{cc} & \;\;\;\;\;\;\text{CTM}\left(r\right)=\frac{\left[\sum_{i=1}^{n-2}\delta \left(d\left(i\right)\right)\right]}{n-2},\\ & \delta \left(d\left(i\right)\right)=\{\begin{array}{cc} 1 & \text{if}\;{\left({\left[x\left(i+2\right)-x\left(i+1\right)\right]}^{2}+{\left[x\left(i+1\right)-x\left(i\right)\right]}^{2}\right)}^{0.5}<r\\ 0 & \text{otherwise}. \end{array} \end{array}$$

**Figure 2:**
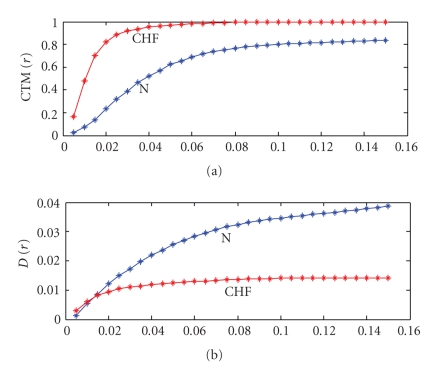
(a) CTM(*r*) versus *r* (b) *D*(*r*) versus *r* for an N (blue) and CHF (red) subject.

For each radius *r*, CTM provides the fraction of the total number of points that lie within it. For a particular radius *r*, CTM counts the number of successive rates that have all sign combinations, without any distinction. Figure 2(a) shows a plot of CTM(*r*) as a function of *r* for a normal and CHF subject, whose SODP is shown in Figure 1. The results of Figure 2(a) show that for this CHF and N subject the radius *r* in the range close to 0.015 provides the best separation in the value of CTM. However the optimum *r* to distinguish between CHF and N is chosen by examining the CTM for a bigger data set of 36 normal and 36 CHF subjects. This is carried out by evaluating CTM(*r*) for the CHF and N subjects and then determining the probability associated with the T statistic that is attributed to the differences in the means of the two data sets being due to chance. The chosen *r *value is the one which gives the smallest probability. This provides the best separation using CTM between the two data sets. This will be done later.

#### 2.2.2. Mean Distance of the Points within the Circular Radius *r*(*D*(*r*))

The mean distance of the points within the circular radius *r* in an SODP is another parameter that is being studied here to characterize the differences between the two distributions. Each point in the SODP is characterized by a distance *d*(*i*) where

(2) $$d\left(i\right)={\left({\left[x(i+2)-x(i+1)\right]}^{2}+{\left[x\left(i+1\right)-x\left(i\right)\right]}^{2}\right)}^{0.5}.$$

The parameter *D*(*r*) is evaluated by determining the mean distance of the points which are within a circular radius *r* *. *The term distance is used here in reference to the SODP plot. Figure 2(b) shows the plot of *D*(*r*) as a function of *r* for the CHF and N subject, whose SODP is shown in Figure 1. The results indicate that significant differences are seen beyond *r* equal to 0.02. The optimum *r* is however chosen using the same procedure used for CTM with the expanded 36 CHF and 36 N subjects.

#### 2.2.3. Component CTM (*C* *C* *T* *M*_*k*_(*r*), k = 1 : 4)

In Section 2.2.1, the fraction of the total number of points that lie within a circular radius *r* in SODP was evaluated. This number involved the counting the number of successive rates that have all sign combinations. On the other hand, the evaluation of  CCTM involves counting the number of points that are present in the four quadrants of the SODP separately, that lie within a circular radius *r*. Let *x* *x*(*i*) = *x*(*i* + 1) − *x*(*i*); *y* *y*(*i*) = *x*(*i* + 2) − *x*(*i* + 1); Then,

(3) $$\begin{array}{cc} \delta \left(d_{1}\left(i\right)\right) & =1\;\text{if}\;\;{\left({\left(xx\left(i\right)\right)}^{2}+{\left(yy\left(i\right)\right)}^{2}\right)}^{0.5}<r,\\ & \;\;\;\;\left(xx\left(i\right)\geq ,yy\left(i\right)>0\right);\\ \delta \left(d_{2}\left(i\right)\right) & =1\;\text{if}\;\;{\left({\left(xx\left(i\right)\right)}^{2}+{\left(yy\left(i\right)\right)}^{2}\right)}^{0.5}<r,\\ & \;\;\;\;\left(xx\left(i\right)<0,yy\left(i\right)\geq 0\right);\\ \delta \left(d_{3}\left(i\right)\right) & =1\;\text{if}\;\;{\left({\left(xx\left(i\right)\right)}^{2}+{\left(yy\left(i\right)\right)}^{2}\right)}^{0.5}<r,\;\;\\ & \;\;\;\;\left(xx\left(i\right)\leq 0,yy\left(i\right)<0\right);\\ \delta \left(d_{4}\left(i\right)\right) & =1\;\text{if}\;\;{\left({\left(xx\left(i\right)\right)}^{2}+{\left(yy\left(i\right)\right)}^{2}\right)}^{0.5}<r,\\ & \;\;\;\;\left(xx\left(i\right)<0,yy\left(i\right)\geq 0\right). \end{array}$$

Otherwise, *δ*(*d*_*k*_(*i*)) = 0, *k* = 1,2, 3,4

(4) $$\text{CCT}{\text{M}}_{k}\left(r\right)=\frac{\left[\sum_{i=1}^{n-2}\delta \left(d_{k}\left(i\right)\right)\right]}{n-2},\;k=1,2,3,4.$$

**Figure 3:**
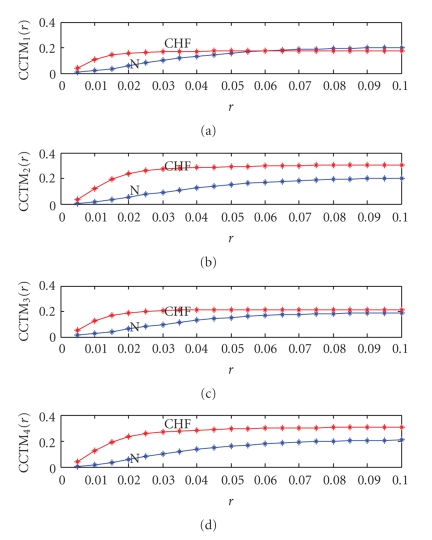
(a) CCTM_1_(*r*) versus *r* (b) CCTM_2_(*r*) versus *r* (c) CCTM_3_(*r*) versus *r* (d) CCTM_4_(*r*) versus *r *for an N (blue) and CHF (red) subject.

In Figure 3  CCTM_*k*_(*r*), *k* = 1,…, 4, are plotted as a function of *r* for both CHF and N subject, whose SODP is shown in Figure 1. The results indicate that the differences are a maximum around *r* in the range of 0.015 to 0.02. Again the optimal radius *r* for each component CTM is chosen using the same procedure used for CTM with the expanded 36 CHF and 36 N subjects.

### 2.3. Classification Systems

Three classification algorithms were investigated. One of them is the *k*-nearest neighbor where the object is classified by a majority vote of its neighbors, with the object being assigned to the class most common amongst its *k* nearest neighbors [11]. In this study *k* = 1, was used, where the subject is assigned to the class of its nearest neighbor. In the *k*-nearest neighbor classification algorithm, both the Euclidean and the Mahalanbois [12] distance were explored. A publicly available matlab code was used for this analysis [13]. The other two classification algorithms studied were the nonparametric tree-based classifier [14, 15] and the support vector machine (SVM) with a linear and a polynomial (degree 2) kernel functions [16–18]. Preliminary investigations showed that the best choice was the *k*-nearest neighbor classification algorithm. The results presented in this paper use this classification algorithm.

### 2.4. Data Used for Analysis

Cardiac RR interval data of 36 normal and 36 CHF patients was used in the analysis. The RR interval data was obtained from MIT-BIH Normal Sinus Rhythm database, BIDMC Congestive Heart Failure database, and congestive heart failure RR interval database posted on Physionet [9]. Before analysis, the raw RR interval time series was preprocessed [10] by the removal of trends and ectopic beats. 

**Figure 4:**
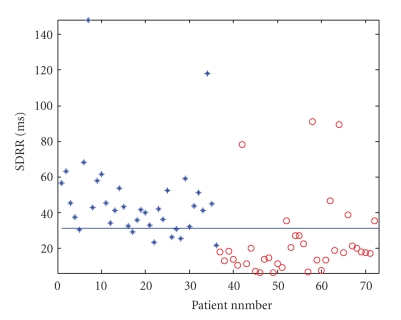
SDRR of normal (∗) and CHF (O) patients. There are 36 N (labeled as 1,…, 36) and 36 CHF (labeled as 37,…, 72).

The ages of the normal subjects in this study were 50.5 ± 17.6 while those of CHF patients were 56.5 ± 11.0. Both groups had both male and female subjects. The majority of the CHF subjects belonged to New York Heart Association (NYHA) classes 3 and 4. There were 2 who belonged to class 2 (patient numbers 63, 65) and 2 in class 1 (patient numbers 62, 64). Subjects classified as belonging to classes 3 and 4 are subjects who suffer from severe congestive heart failure. Subjects in class 2 show mild limitation of activity while class 1 suffers no symptoms from ordinary activities [19]. A plot of the standard deviation of the RR intervals (SDRR) in milliseconds is shown in Figure 4 for the 36 normal and 36 CHF subjects. The blue line drawn is a suitable threshold [20] drawn to separate N from CHF. The number of RR intervals used here is 70000.

## 3. Results and Discussion

In this section the SODP features discussed earlier are evaluated for the 36 normal and 36 CHF patients using the RR interval cardiac data. Also included in this section are the results of SDRR. For each of these 36 N and 36 CHF subjects CTM(*r*), *D*(*r*), CCTM_1_(*r*), CCTM_2_(*r*), and CCTM_3_(*r*), CCTM_4_(*r*) are obtained from their SODP for different *r* values. The values of *r *used were in the range shown Figures 2 and 3. For each of these *r* values the *t*-test at significance level of 0.05 was performed to determine whether the samples from the normal and CHF distribution have the same mean. The result is given in terms of values for *h*, *p*, and ci. A value of *h* equals to one indicates that one can reject the null hypothesis at the 0.05 significance level. The *p* value indicates the probability of observing a *t* value $(=\left(\bar{x}-\bar{y}\right)/s$ where $\bar{x\;}$ and $\bar{y}$ are the mean values of normal and CHF subjects and *s* the pooled standard deviation) as large or larger by chance under the null hypothesis. A low value for *p* smaller than the significance level indicates that the null hypothesis is improbable. The ci range indicates the 95% confidence interval of the true difference in means. If it does not include zero, it indicates that there is a difference. In Table 1, the *r * that gives the lowest value of *p* for the range of *r* values studied are given for each of these measures, along with values of ci. The value of *h* for all these reported cases is 1, implying that there is difference in the means at 95% confidence level. Also included in this table are the *p* and ci values for SDRR. The value of *r* given in table is not the only *r* value where the means are significantly different. There are a number of other *r* values where *h* = 1. The *r *value given is the one which has the smallest *p* in the range of *r* values tested here. This gives the best separation between the N and CHF subjects. The number of points (*n*) used is 30000. The table also includes values of *p* and ci for this *r* value for SODP plots where the number of points has been increased to 70000. In the last row of this table, the *p* and ci values for SDRR are shown for *n* = 30000 and 70000. The *p* values for SDRR are much greater compared to the measures used for SODP. The results for *n* = 70000 show that better distinction between N and CHF is obtained compared to *n* = 30000. Since they provide a better separation, the features from this data set will be used for training. 

The results of Table 1 clearly show that all the 6 features of the SODP exhibit significant differences between the normal and CHF subjects. Next we attempt to use these measured features of these SODP for classification. These 6 features of the SODP can be used alone or in groups of 2, 3, 4, 5, and 6. A total of 63 possible groups are possible using the measured 6 features. All these feature groupings are used in the *k*-nearest neighbor classifier algorithm to examine the performance of this scheme to classify the SODP features of an unknown subject. The classification scheme requires a training set. Suppose that we use our test data (the feature data set which we want to classify as either N or CHF), as one of the SODP feature sets from the 36 N and 36 CHF subjects. The training set that is used in this case is the SODP features of the 71 subjects that exclude the test data. The feature set used for the training data is the SODP features obtained from the RR intervals where *n* = 70000. This set as seen from table provided the best separation between N and CHF patients. For the test data the SODP features using 30000 RR intervals is used. Instead of using the full data set of *n* = 70000 for the test data we use a shorter RR interval data set, so that classification can be carried out many times with different data sets of the same subject. This is done in order to minimize random errors in the algorithm. Thus the algorithm is repeated with different feature sets of the same subject obtained from different sets of 30000 RR intervals, within the *n* = 70000 RR intervals. These different 30000 RR interval data sets start at various positions within the 70000 data set. The classification algorithm is run for each of these test data which are different realizations of the feature set for the same subject. Suppose that the number of these different realizations is *mc. *In this study we had *m* *c* = 31. In every classification run the subject *i* is assigned a value of 1 for *p*_*i*_(*l*) if the subject is misclassified, and 0 when the classification is correct.

(5) $$\begin{array}{cc} P\left(i,j\right) & =1\;\text{if}\;\;\overset{mc}{\underset{l=1}{\sum }}\frac{p_{i}\left(l\right)}{mc}>0.95,\\ P\left(i,j\right) & =0\;\text{otherwise}. \end{array}$$

Here *i* refer to the subject and *j* to the feature set group. The above result is for a particular feature grouping. Suppose that we have fg feature groups. For example, if we use 6 features, then we can have 63 possible groups. Thus fg = 63. If we use only two of them then fg = 3. If only one feature is used fg = 1. The subject *i* is misclassified if

(6) $$\overset{\text{fg}}{\underset{j=1}{\sum }}\frac{P\left(i,j\right)}{\text{fg}}>0.95.$$

Thus to minimize random errors in classification, multiple runs of the classification algorithm are carried out with feature sets from different sets RR intervals of the same subject, and different groupings of the feature sets. Table 2 show that the results of this study for 36 N and 36 CHF subjects. The results are shown for the two distances Euclidean and Mahalanbois. 

The results of Table 2 showthat the *k*-NN classification system using features of the SODP obtained from RR intervals has performed very well, with 100% success rate, when the six feature set is used. In this case, the result is the same for both Euclidean and Mahalanbois distance measures. For the two feature set, the performance is better if the Mahalanbois distance measure is used. The Mahalanbois distance, unlike the Euclidean distance which depends only on the distance between two vectors, takes into account the correlation of the other vectors present. It is a statistical distance, and requires the covariance matrix of the vectors present. One would therefore expect that this classification system using this will be better than the Euclidean distance. This is seen here for the two feature set of the SODP. In fact a simpler two feature set of {CTM(*r*), *D*(*r*)} gives zero misclassification using the Mahalanbois distance. The performance using SDRR is also good, where only one subject is misclassified. If the performance of the classification system using SDRR is compared with the simple threshold used in Figure 4, it is clear that this statistical approach provides a far superior result, instead of a using a simple threshold. In Figure 4, thirteen subjects are misclassified, which reduces to one in the new scheme. The only subject misclassified belongs to class 1, which shows no symptoms from ordinary activities [19].

## 4. Conclusion

In this paper a classification scheme to separate N from CHF subjects is studied using the RR intervals obtained from the Holter monitor. This is carried out using the features from a second-order difference plot obtained from the RR intervals. The RR interval data was preprocessed to remove trends and any ectopic beats present in the time series before second-order difference plots were drawn and several features extracted. Six features were obtained from the second-order difference plot. These features are central tendency measure, mean distance of the points within the circular radius *r*; and the four component central tendency measures. These features are then used as input into a *k*-nearest neighbor algorithm, with *k* = 1, for classification. The final classification result is obtained using a statistical procedure which involves running the classification algorithm many times with different feature sets of the same subject and different groupings of the features. This has the effect of reducing random errors. The study focused on determining whether a patient is healthy or has congestive heart failure from the Holter monitor RR interval data. For this study the Holter monitor data of 36 normal and 36 CHF patients was used. The result of this study showed a 100% classification rate using the features obtained from the second-order difference plot. 

The study also showed that the standard deviation of RR intervals also performed well using this procedure, although it could not reach the 100% success rate achieved using the features from the second-order difference plot. However the success rate for classification was many fold higher compared to the simple procedure of using a threshold. The results of the classification procedure using both the SODP features and SDRR are encouraging and one would expect such consistent results with the use of larger training sets. The analysis outlined in this paper will be a valuable asset to the clinician, in addition to the clinical and history information of the subject, to provide a useful strategy in the detection congestive heart failure.

## Figures and Tables

**Table 1:** *T* test results for CTM(*r*), *D*(*r*), CCTM_1_(*r*), CCTM_2_(*r*), CCTM_3_(*r*), CCTM_4_(*r*), and SDRR. The *r* value corresponds to the lowest value of *p* for each of these measures.

Measure used	*r *	*p* (30000)	*p* (70000)	ci (30000)	ci (70000)
CTM(*r*)	0.015	6.63	7.5688	[−0.3666 −0.2067]	[−0.3965 −0.2415]
		(−10)	(−12)		
*D*(*r*)	0.035	3.4314	3.1584	[0.0039 0.0063]	[0.0043 0.0066]
		(−12)	(−14)		
CCTM_1_(*r*)	0.015	3.5834	2.3475	[−0.0741 −0.0380]	[−0.0808 −0.0465]
		(−8)	(−10)		
CCTM_2_(*r*)	0.015	4.5038	1.1636	[−0.1004 −0.0543]	[−0.1102 −0.0642]
		(−9)	(−10)		
CCTM_3_(*r*)	0.015	1.9055	2.5181	[−0.0972 −0.0536]	[−0.1015 −0.0607]
		(−9)	(−11)		
CCTM_4_(*r*)	0.015	3.5953	1.1240	[−0.1002 −0.0545]	[−0.1094 −0.0638]
		(−9)	(−10)		
SDRR		3.7432	6.0420	[0.0092 0.0303]	[0.0123 0.0339]
		(−4)	(−5)		

**Table 2:** Final classification results for different feature sets and distance measures.

Feature set used	No. of	No. of misclassified
	misclassified	patients
	patients	(Mahalanbois
	(Euclidean distance)	distance
CTM(*r*)	2	2
{CTM(*r*), *D*(*r*)}	1	0
{CTM(*r*), *D*(*r*)	0	0
CCTM_1_(*r*), CCTM_2_(*r*)		
CCTM_3_(*r*), CCTM_4_(*r*)}		
SDRR	1	1
